# Medium- and long-term recurrence after radioiodine therapy for differentiated thyroid carcinoma with recombinant human thyrotropin: a meta-analysis

**DOI:** 10.3389/fendo.2024.1474121

**Published:** 2024-12-17

**Authors:** Qixian Yao, Lili Song, Jun Xu, Zhongliang Wu

**Affiliations:** ^1^ Department of Surgery, Community Health Service Center of Suzhou Science & Technology Town, Suzhou, Jiangsu, China; ^2^ Department of General Surgery, The Second Affiliated Hospital of Soochow University, Suzhou, Jiangsu, China; ^3^ Department of Rehabilitation, The People’s Hospital of Suzhou New District, Suzhou, Jiangsu, China

**Keywords:** recombinant human thyroid stimulating hormone, differentiated thyroid carcinoma, long-term recurrence, thyroid hormone withdrawal, radioactive iodine

## Abstract

**Introduction:**

Radioactive iodine (RAI) is commonly used in the management of differentiated thyroid cancers (DTCs). However, the long-term efficacy and the risk of tumor recurrence associated with it remain unclear. In particular, the comparison between recombinant human thyrotropin (rhTSH) and thyroid hormone withdrawal (THW) in terms of medium- and long-term recurrence rate in DTC patients has not been fully elucidated.

**Methods:**

A systematic search was carried out to identify articles comparing medium- and long-term outcomes (> 2 years) based on treatment with either rhTSH or THW. Ten studies, consisting of six randomized controlled trials (RCTs) and four retrospective studies with a total of 2,833 patients, were included in the analysis.

**Results:**

There was no significant difference in the medium- and long-term recurrence rates between the rhTSH group and the THW group. This was also the case in subgroup analyses of only RCTs or only retrospective studies. The structural incomplete response (SIR) rate was slightly higher in the rhTSH group, but a subgroup analysis of RCTs alone showed no significant difference in SIR between the two groups.

**Discussion:**

rhTSH is comparable to THW in achieving successful ablation of residual disease and maintaining low recurrence rates. However, further RCTs are required to investigate whether rhTSH can increase the risk of SIR.

## 1 Introduction

Thyroid cancer is the most common endocrine tumor, and its incidence has been increasing globally in recent decades (1). Approximately 614,000 and 206,000 new cases in females and males, respectively, were reported worldwide in 2022 (2). The most common type, differentiated thyroid cancer (DTC), constitutes about 90% and has a good prognosis (3). With standardized treatment, clinical remission, which has a 10-year cancer-specific mortality rate of less than 10%, is typically achievable. However, when distant metastasis occurs, the overall survival rate of DTC decreases to 40% (4). The main treatment modality for DTC includes total or near-total thyroidectomy, radioactive iodine (RAI) therapy, and thyroid hormone suppression therapy for at least six decades (5). RAI treatment refers to the clinical use of iodine-131 (^131^I), a radioisotope of iodine. This practice is widely accepted in the management of DTC and is often used post-surgery for the ablation of residual thyroid tissue (6). For RAI to be effective, a heightened concentration of thyroid stimulating hormone (TSH) is necessary to maximize the uptake of ^131^I in normal or neoplastic thyroid cells. This can be achieved through thyroid hormone therapy withdrawal (THW) or the administration of recombinant human TSH (rhTSH) (5).

The European Medical Association first approved rhTSH in 2005 to be used as an adjunctive tool of RAI for diagnostic or therapeutic purposes in patients who have undergone thyroidectomy for DTC (7). This was followed by an FDA approval in 2007. A meta-analysis and multiple other studies have confirmed that the ablation rate of residual tissue after rhTSH administration is similar to that obtained after THW (8–11). In contrast to THW, patients can continue levothyroxine (LT4) supplementation, which aids in avoiding short-term hypothyroidism, preserving quality of life, maintaining normal renal iodine clearance, and reducing potential side effects (12). Therefore, rhTSH has garnered recommendations in multiple guidelines for use alongside RAI in patients with DTC (5, 13).

Despite the long-term clinical use of rhTSH, most studies have focused on its short-term efficacy. Hence, there is still a lack of research on long-term efficacy and recurrence, a gap that persists even in the most recent guidelines (13). This meta-analysis summarized previous studies, with the main objective of evaluating the long-term efficacy of RAI therapy using various preparation methods. The overarching goal was to aid decision making based on the efficacy results.

## 2 Methods

This meta-analysis follows the Preferred Reporting Items for Systematic Reviews and Meta-Analyses (PRISMA) statement and is registered in the PROSPERO database (CRD42023437018).

### 2.1 Search strategy

Two investigators (L.S. and Z.W.) performed independent searches in electronic databases, including PubMed, Embase, Cochrane Library, Web of Science, and ClinicalTrial, for articles published in English from inception until May 2023. There was no restriction based on country of origin or article type. The reference list of each article was independently screened to identify additional studies not obtained from the initial search. The following search terms were used: “thyrotropin alfa,” “rhTSH,” “thyroid neoplasms,” “follow-up,” and “long-term.”

### 2.2 Study selection and inclusion/exclusion criteria

Studies were screened for appropriateness before retrieval of the full article by two reviewers (Q.Y. and L.S.), and disagreements were resolved by consensus. Studies were included for analysis if all the following criteria were met:

(1) Randomized controlled clinical trials (RCTs) or observational studies with a retrospective or prospective design that evaluated patients with DTC;
(2) Studies evaluating clinical outcomes from adult patients (at least age 16 years);
(3) Patients must have undergone total or near-total thyroidectomy as a primary treatment for DTC;
(4) Median duration of follow-up of more than 2 years.

### 2.3 Risk of bias and quality assessment

Each eligible RCT was assessed by two independent reviewers (Q.Y. and L.S.) using the Cochrane risk of bias tool 2.0, which reviewed the following seven areas: random sequence generation, allocation concealment, masking of participants and personnel, masking of outcome assessment, incomplete outcome data, selective reporting, and other biases (14). The quality of observational studies was also assessed using the Newcastle-Ottawa scale (15). A risk of bias graph was drawn, and a risk of bias summary was compiled.

### 2.4 Statistical analysis

Statistical analysis was performed using the Review Manager software (version 5.3; The Nordic Cochrane Center). Forest plots were generated for dichotomous variables. Dichotomous variables were analyzed using the Mantel-Haenszel risk ratios (RRs) with 95% confidence intervals (CIs), while continuous variables were analyzed using weighted-mean differences. Heterogeneity between studies was assessed using the Cochrane χ*2* value, and the degree of heterogeneity was measured by the *I^2^* value. A fixed-effects model was used for calculations unless significant heterogeneity existed (*I^2^* >50%), in which case a random-effects model was used. A *P* value of <0.05 was considered statistically significant. Sensitivity analysis was designed to exclude one study at a time, RCTs or observation studies only, studies with a long median follow-up time (>5 years), and studies after 2015. Secondary outcomes were considered successful ablation, structure incomplete response, and biochemical incomplete response (5).

## 3 Results

### 3.1 Findings from the literature search

**Figure 1:**
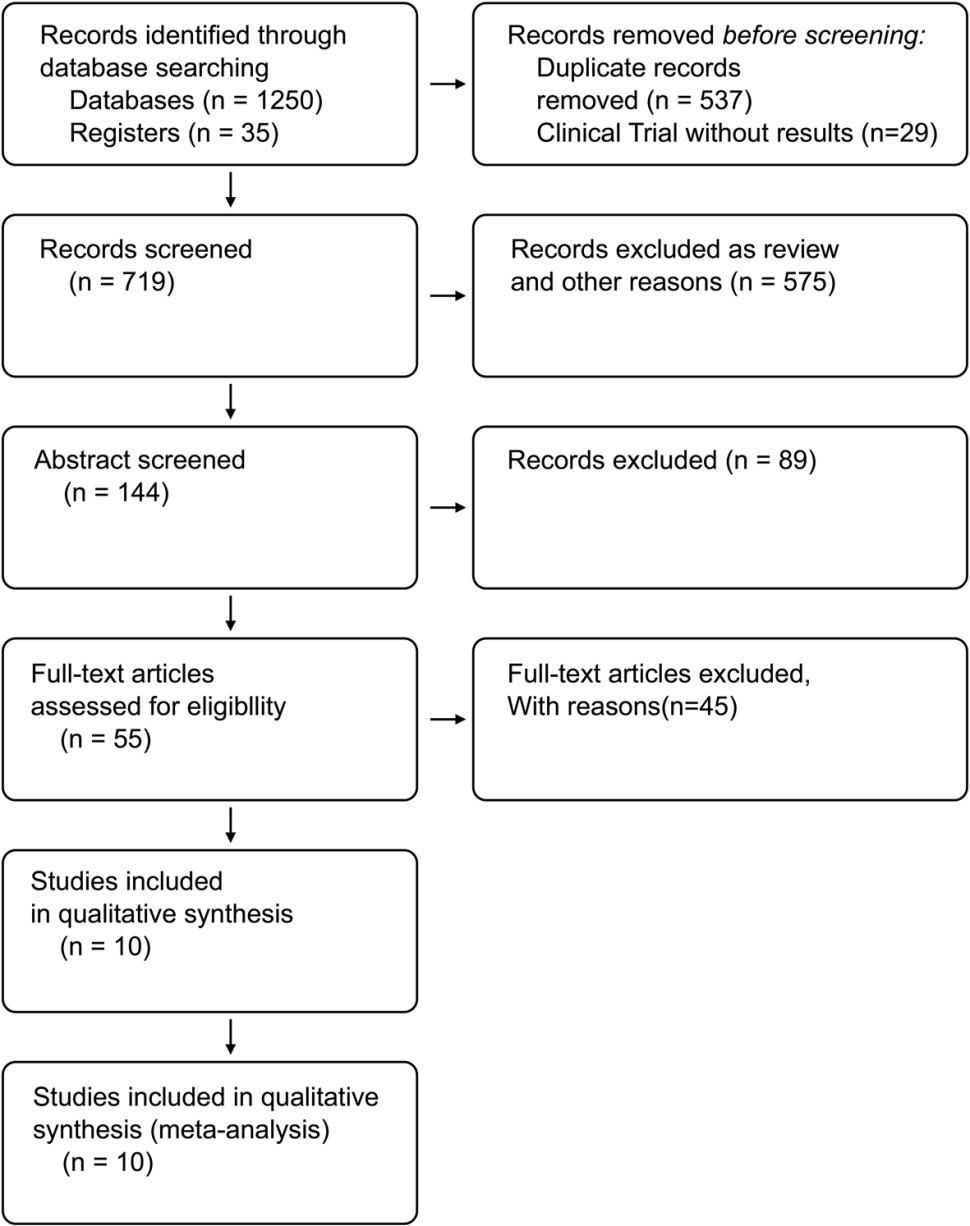
Flow diagram showing study selection for the systematic review and meta-analysis.

A total of 1,285 studies were identified following the comprehensive search, of which 1,230 were excluded based on titles and abstracts. A total of ten studies were considered eligible, including six RCTs (16–21) and four retrospective studies (22–25). **Figure 1** shows the flowchart of our search strategy.

### 3.2 Study characteristics

The ten included studies recruited 2,833 patients and were conducted in Europe, South America, Canada, and the US. Doses of ^131^I ranged from 30 mCi to more than 200 mCi. One study focused on patients with low-risk DTC (16), and another examined patients with intermediate-risk DTC (19). Additionally, several other studies investigated patients across multiple risk levels of DTC. The median follow-up time was at least 3.7 years (16). The characteristics of the included RCTs and observational studies are illustrated in **Table 1**.

**Table 1:** Characteristics of the included studies.

Source	Type	Participants	Area	Age, years	Sex, Female	Pathology, PTC	TNM Stage	Radio Activity	Follow-up	Criteria for no recurrence	Risk Categor(ies)
Ladenson et al. (2009) (16)	RCT	51	USA, France, Italy, Germany, Canada	48 ± 12*	41	45/6	T1/2/4 N0-1 M0	100mCi	3.7 y^$^	No visible uptake	L
Rosario et al. (2012) (17)	RCT	276	Brazil	rhTSH 45.4 ± 9.2	192	253	T1-3 N0-1	30, 100 or 150 mCi	70 mon^$^	Negative imaging, cytology or histology, and/or no unequivocal ectopic uptake on WBS	L, H
				THW 46.1 ± 9.6							
Elisei et al. (2013) (18)	RCT	120	Italy	UK	79	113	T1-4 N0-1 M0	30mCi	At least 10 y	Without persistent disease	L, M, H
Rosario et al. (2016) (19)	RCT	178	Brazil	rhTSH 47^$^	134	178	N1b	30, 50 or 100 mCi	66 mon^$^	Negative imaging, cytology, or histology, and/or no unequivocal ectopic uptake on WBS or PET-CT	M
				THW 48^$^							
Schlumberger et al. (2018) (20)	RCT	726	France	UK	UK	669	T1-2 N0-1/X	30 or 100mCi	5.4 y^$^	Suppressed Tg ≤1 ng/mL and normal results on neck US	L, M, H
Mallick et al. (2019) (21)	RCT	434	United Kingdom	from 18 to 82	326	229	T1-3 N0-1	30 or 100mCi	78.4 mon^$^	Negative in Tg, US, aspiration or biopsy, and imaging	L, M
Tamilia et al. (2016) (22)	Retro	370	Canada	55.2 ± 12.4	302	161	T1-3 N0-1/X	30mCi	rhTSH 7.1 y ^$^ THW 9.3 y	Negative in suppressed Tg, TgAb, and neck sonography	L, M
Pitoia et al. (2016) (23)	Retro	219	Argentina	rhTSH 48.7 ± 13.2	170	219	UK	<100 – >200mCi	rhTSH 64.5 mon ^$^	Suppressed Tg <1ng/ml, with TgAb negative, and negative in neck US, imaging, or any pathological findings	L, M, H
				THW 42.9 ± 12.1					THW 55 mon		
Leenhardt et al. (2019) (24)	Retro	404	France	rhTSH 46.0 ± 15.2	301	396	pT1-T3 N1 M0	3.27 ± 1.00 GBq	rhTSH 29.68 ± 20.73 mon	Complete remission	UK
				THW 45.6 ± 14.7					THW 36.66 ± 23.83 mon		
Burman et al. (2022) (25)	Retro	55	USA	rhTSH 41^$^	39	40	M1	rhTSH 329 ± 197MBq	rhTSH 4.2 y^$^	UK	H
				THW 59^$^				THW 418 ± 228Mbq	THW 6.8y ^$^		

*Age at remnant ablation.

^$^Results showed by the median.

Retro, retrospective study; RCT, randomized clinical trial; Tg, serum Thyroglobulin; Dx-WBS, diagnostic radioactive iodine scans; TgAb, Tg antibodies; US, ultrasound; PTC, papillary thyroid cancer; H: high-risk; M: intermediate-risk; L: low-risk; UK, unknown.

### 3.3 Patients’ characteristics

The rhTSH group included 1,303 participants (46%), and the THW group included 1,530 (54%) participants. There were 1,584 and 513 female and male participants, respectively, and the gender of the remaining 726 participants was not mentioned (20). A total of 2,303 participants (81.3%) received a diagnosis of papillary thyroid cancer (PTC), and 530 (18.7%) had other subtypes of thyroid cancer.

### 3.4 Quality assessment

**Figure 2:**
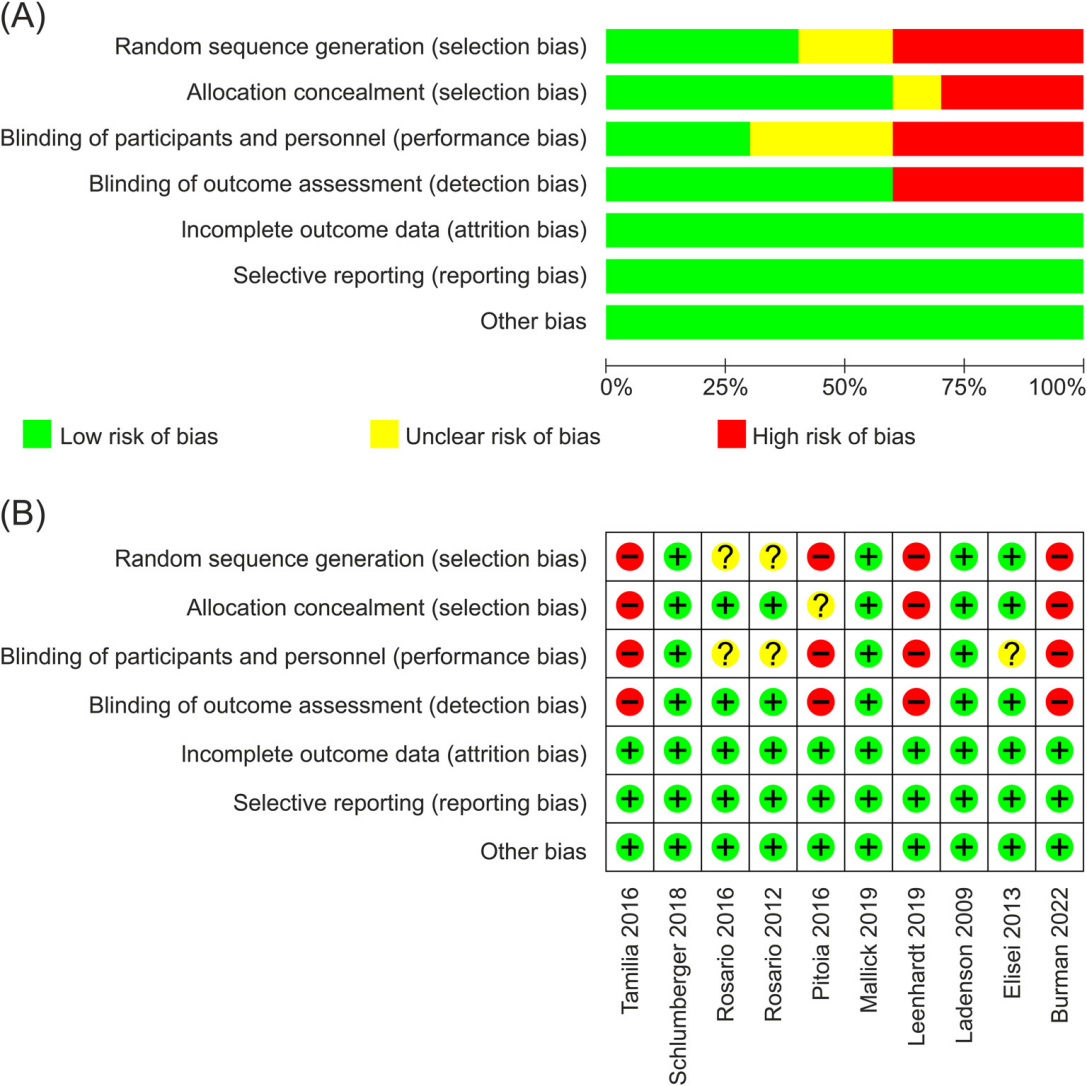
**(A)** Risk of bias graph. **(B)** Risk of bias summary.

The methodological quality of the studies assessed with Newcastle-Ottawa scale was generally good. **Figures 2A, B** provide a risk of bias summary and graph for the included studies. Studies were all open-label because of different methods to stimulate TSH in two groups (the rhTSH group using rhTSH for two consecutive days and the THW group discontinuing thyroid hormone for 3–6 weeks). Three studies employed independent imaging reviews to exhibit an approach aimed at minimizing bias resulting from the non-blind nature of all included studies (16, 20, 21).

### 3.5 Primary outcomes

#### 3.5.1 Recurrence rates

**Figure 3:**
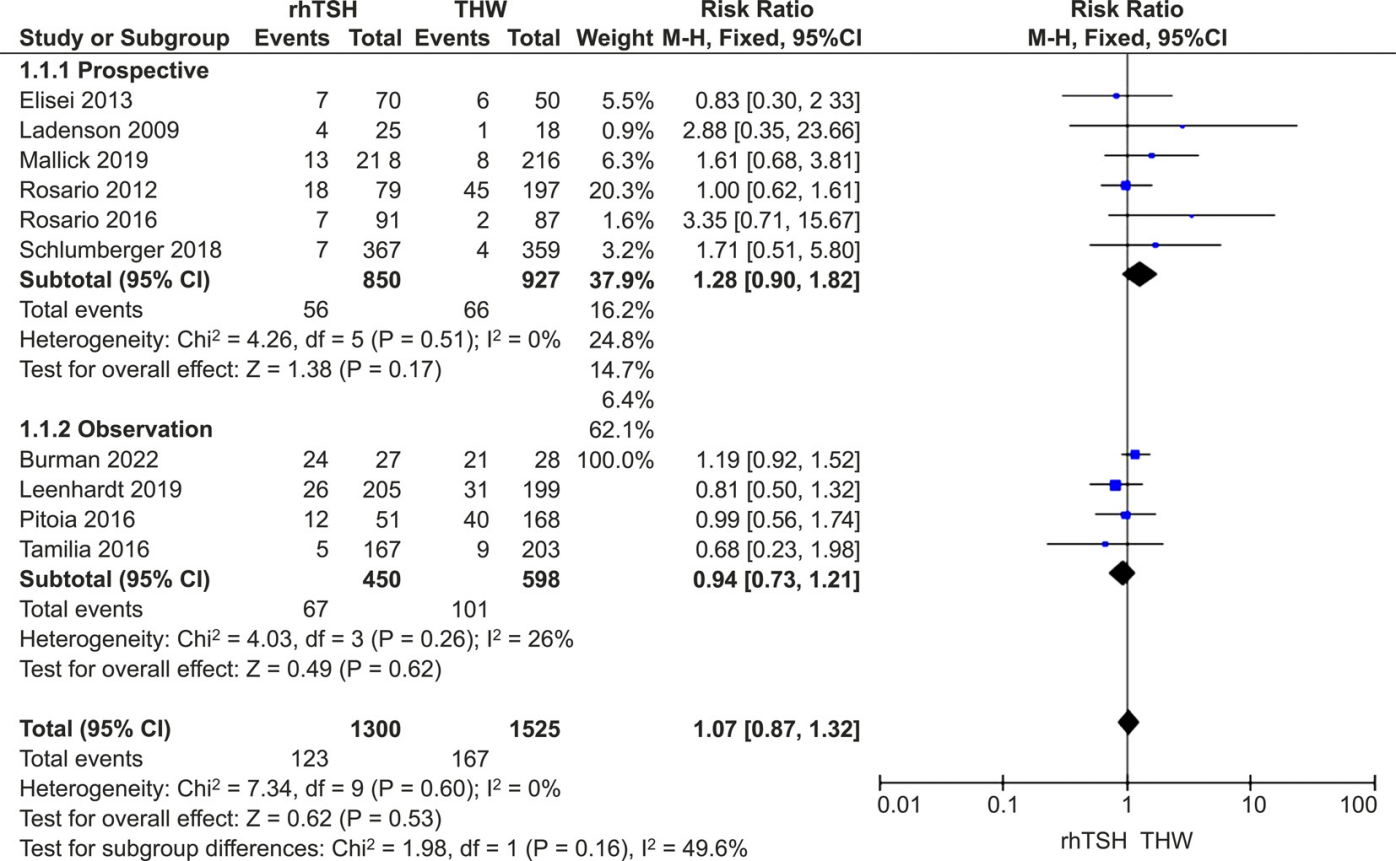
Forest plots comparing the recurrence rates between groups receiving rhTSH and THW, including subgroup analyses of RCTs and retrospective studies. rhTSH, recombinant human thyroid stimulating hormone; THW, thyroid hormone withdrawal; RCT, randomized controlled trial.

One hundred and twenty-three participants (9.5%) experienced recurrence in the rhTSH group, while 167 individuals (11.0%) in the THW group had a recurrence. There was no heterogeneity among the included studies (*I* ^2^ = 0%; *P*=0.68), so a fixed-effects model was used. No statistical difference in recurrence rates was observed between the two groups (RR=1.07; 95% CI, 0.87–1.32; *P* = 0.53) (**Figure 3**).

#### 3.5.2 Sensitivity analysis and subgroup analysis based on study design

Exclusion of any study did not significantly alter the findings from the main analysis. Analysis of only RCTs (RR=1.28; 95% CI: 0.90–1.82; *P*=0.17) or observation studies (RR=0.94; 95% CI: 0.73–1.21; *P*=0.62) showed no difference in recurrence rates between the groups (**Figure 3**).

#### 3.5.3 Subgroup analysis of studies with 5-year follow-up

Seven studies provided long-term (at least 5-year) follow-up data (16, 17, 19–23). There was no difference in recurrence rates between the two groups in this subset of studies (RR=1.14; 95% CI, 0.81–1.60; *P* = 0.46; *I* ^2^ = 0%; *P*=0.60).

#### 3.5.4 Subgroup analysis of studies conducted after 2015

Seven studies conducted after 2015 were included in our analysis (19–25). There was no difference in recurrence rates between the two groups in this subset of studies (RR=1.12; 95% CI, 0.80–1.56; *P* = 0.51; *I* ^2^ = 14%; *P*=0.32).

#### 3.5.5 Subgroup analysis based on ^131^I dose

**Figure 4:**
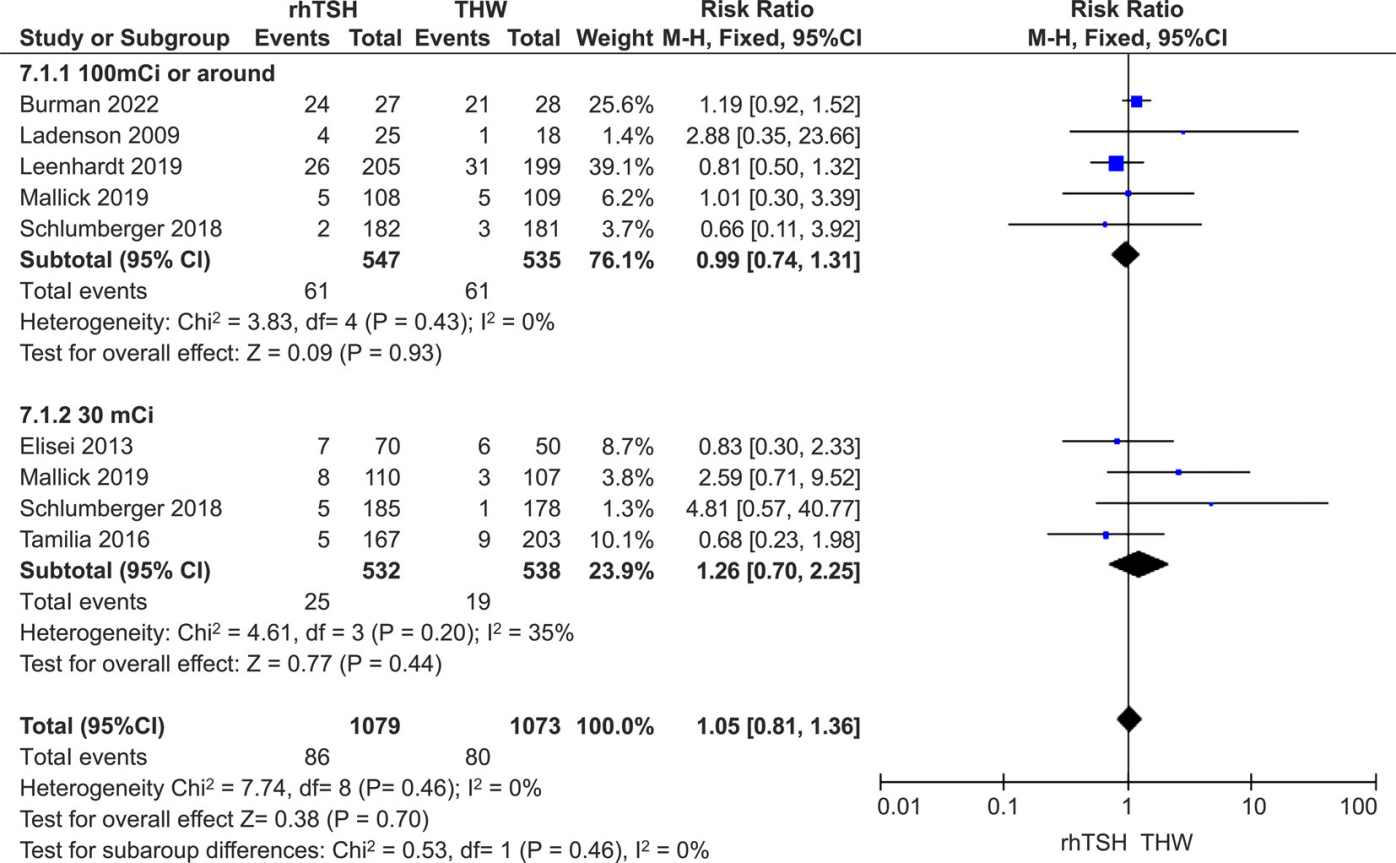
Subgroup analysis based on different ^131^I doses.

Five studies provided medium- and long-term follow-up data on patients who received approximately 100 mCi of RAI (16, 20, 21, 24, 25), and four studies provided data for those who received approximately 30 mCi (18, 20–22). The other studies did not clearly present the situation of patients with different doses (17, 19, 23). There was no difference between the two groups in this subset of studies (RR=1.05; 95% CI, 0.81–1.36; *P*=0.46; *I* ^2^ = 0%; *P*=0.46) (**Figure 4**).

#### 3.5.6 Subgroup analysis based on risk strata

Only three studies provided data on low-risk patients (16, 18, 23), while four studies provided detailed data on intermediate- and high-risk patients (16, 17, 23, 25). Other studies did not detail the specific data of each risk level (19–22, 24). There was no significant difference (RR=1.33; 95% CI, 0.96–1.84; P=0.09; *I^2^ =* 45%; P=0.12). Furthermore, detailed data showed only 229 low-risk patients and 337 intermediate- and high-risk patients, so the results may not be reliable.

### 3.6 Secondary outcomes

#### 3.6.1 Successful ablation

Nine studies were included in this analysis (16–24), and the rate of successful ablation was similar in both groups (80.5% vs. 81.2%). Our meta-analysis demonstrated no significant difference in successful ablation between studies that utilized different preparations for RAI (RR=0.81; 95% CI, 0.66–1.00; *P*=0.05; *I* ^2^ = 0%; *P*=0.75). In an analysis of RCTs only, there was no significant intergroup difference in ablation rates (RR=0.88; 95% CI, 0.65–1.20; *P*=0.41).

#### 3.6.2 Structural incomplete response

**Figure 5:**
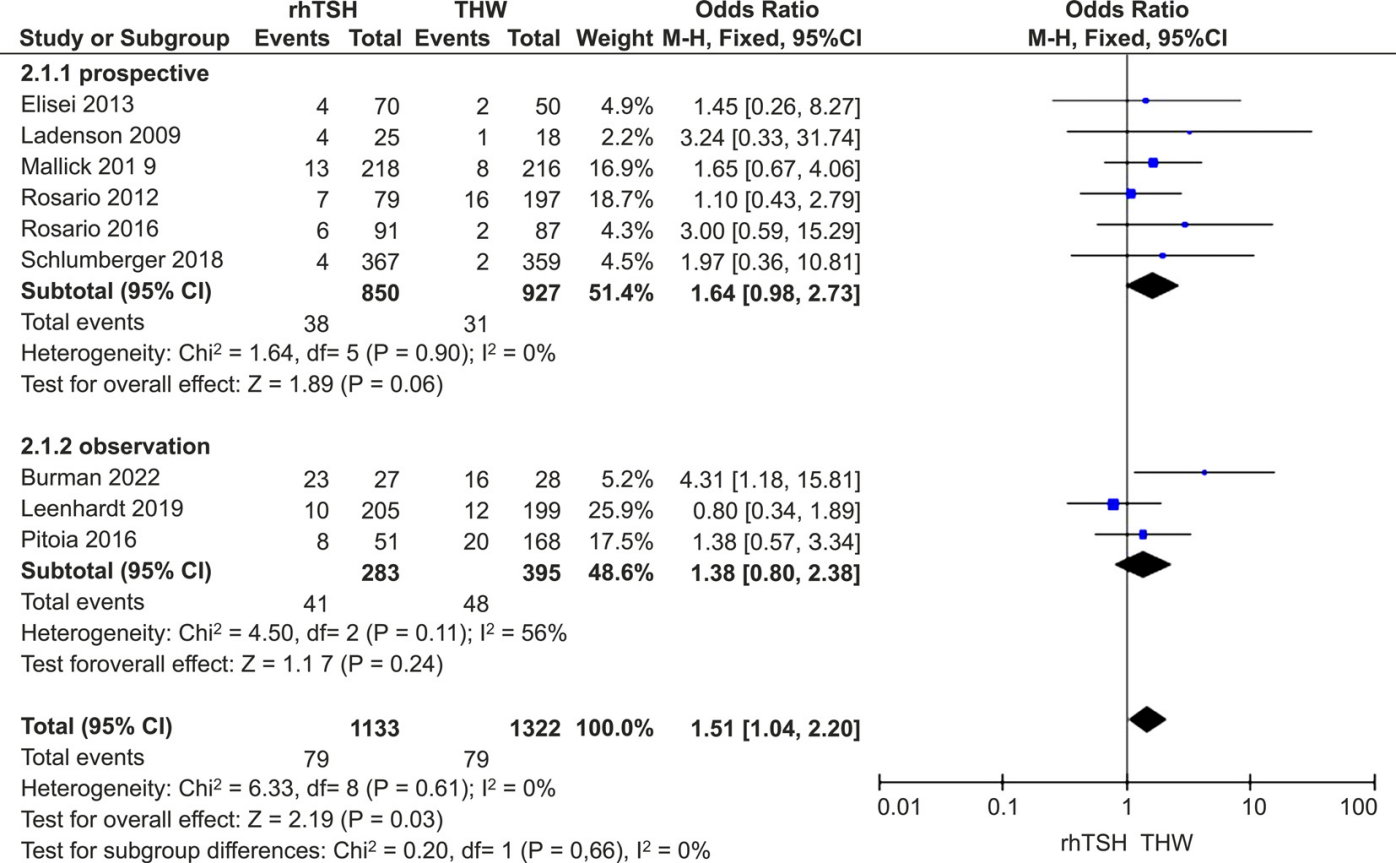
Forest plots comparing SIR rates between groups receiving rhTSH and THW, including subgroup analyses of RCTs and retrospective studies. SIR, structural incomplete response; rhTSH, recombinant human thyroid stimulating hormone; THW, thyroid hormone withdrawal; RCT, randomized controlled trial.

There was no heterogeneity of studies (*I* ^2^ = 0%; *P* = 0.61); hence, a fixed-effects model was used. Our meta-analysis of nine studies (16–24) demonstrated that the SIR rate was a little higher in the rhTSH group (RR=1.51; 95% CI, 1.04–2.20; *P*=0.03). However, a subgroup analysis of RCTs showed no significant difference between the two groups (RR=1.64; 95% CI, 0.98–2.73; *P*=0.06) (**Figure 5**).

#### 3.6.3 Biochemical incomplete response

This analysis included five studies (16, 20, 23–25) with no heterogeneity (*I* ^2^ = 0%; *P*=0.61). Meta-analysis demonstrated no significant difference between the two groups of participants (RR=0.76; 95% CI, 0.47–1.25; *P*=0.28). Only two RCTs provided BIR data, so the analysis of RCTs seemed insignificant (16, 20).

## 4 Discussion

For over two decades, rhTSH has been available and included in recommendations by several guidelines to replace THW for use alongside RAI (5, 13). However, some issues regarding its use require further clarification. One is the medium- and long-term efficacy of RAI combined with rhTSH. DTCs are known to grow slowly and have a good prognosis, with a 5-year overall survival rate of over 95%, while the recurrence rate ranges from 14% to 30%. Therefore, the risk of recurrence and the medium- and long-term persistence of DTC are worthy of attention. However, the long-term follow-up data with a duration exceeding 5 years is relatively scant. Two years can act as an intermediate milestone to preliminarily gauge the treatment effectiveness. Future research will be dedicated to obtaining more long-term follow-up data to more accurately assess the recurrence situation. The most important finding from our meta-analysis comparing the medium- and long-term efficacy of rhTSH and THW in patients undergoing RAI for DTC showed no difference between the two methods, and this was consistent with the results of RCTs.

Notably, the definition of recurrence adopted in various studies was inconsistent. Some articles defined recurrence as “clinical evidence of tumor recurrence after a disease-free period, such as suspicious lymph nodes, thyroid bed nodules, or highly suspicious metastatic lesions,” which is similar to SIR (20, 21). Other studies added biochemical indicators for recurrence assessment (16, 23), although the criteria were disparate. This limitation and discrepancy in the employed criteria affected recurrence results.

A release by the American Thyroid Association in 2015 (ATA 2015) advocated for tailored management of DTC based on prognosis. The guideline provided evidence that tumors identified with RAI imaging can be used as criteria for structural assessment and detection of stimulating thyroglobulin levels (> 1ng/mL) for biochemical recurrence. The disease outcome at any point during follow-up in the guideline is described by four categories: excellent response (ER), BIR, SIR, and indeterminate response (IDR). This guideline also provided a favorable evaluation framework rather than methods to evaluate the prognosis of DTC, which is a more convenient and accurate approach. Of these four outcomes, BIR has a 20% probability of progressing into SIR (26, 27). Also, 50% to 85% of patients with SIR continue to have persistent disease (28–30), further lending credence to the fact that these two outcomes warrant attention. According to the specific situation of disease recurrence, SIR and BIR were analyzed in this meta-analysis. The results showed that the SIR rate was slightly higher in the rhTSH group than in the THW group, with similar results in RCTs, while there was no difference in the BIR rate of both groups. SIR deserves attention in clinical practice because it indicates a poor prognosis. The results of this meta-analysis show that the medium- and long-term outcomes of rhTSH in SIR require further investigation. More RCTs may be needed to confirm the specific results.

Due to the importance of the ATA 2015 guideline, a subgroup analysis of studies published after 2015 was conducted. Additionally, the guideline clarified that low-risk patients can be exempted from RAI treatment, and a subgroup analysis of different patient categories was also conducted. The results of these two analyses showed no significant difference between patients receiving rhTSH and THW. However, it is worth noting that no RCTs were launched after 2015, and the statistics of intermediate- and high-risk patients were unclear in the studies of this meta-analysis. This constitutes a major limitation of our study.

The participants included in this study were mainly intermediate-risk and low-risk patients, with a few being high-risk. It is not clearly indicated whether rhTSH can be administered to high-risk patients. Several retrospective studies have shown that for ^131^I adjuvant therapy, the therapeutic efficacy and medium- and long-term prognosis observed in the rhTSH and THW groups are similar (31, 32). However, no prospective studies have corroborated this finding. For the risk analysis, we acknowledge that the limited number of low-risk patients (only 229 cases) and the unclear statistical data of intermediate- and high-risk patients in the subgroup analysis are indeed limitations of this study. In future research, we plan to more strictly define the risk criteria and increase the sample size, especially for medium- and long-term follow-up studies of patients at different risk levels, to improve the reliability of the risk analysis.

The “Martinique Principles” propose that ^131^I therapy can be used for residual ablation, adjuvant treatment, and treatment of known disease (33). They also recognize that residual ablation and adjuvant treatment cannot be accurately distinguished. Otherwise, subgroup analysis based on the dose makes the comparison among different studies more feasible and meaningful as the dose is a more relatively objective and quantifiable indicator than indication. Therefore, we conducted a subgroup analysis based on the dose of ^131^I rather than the indication of use (residual ablation or adjuvant treatment). Although the difference is not significant, the choice of ^131^I dose remains a debatable issue, particularly in situations where residual ablation is recommended only in intermediate- and high-risk patients and also combined with adjuvant treatment. More studies specifically focusing on medium- and long-term recurrence with different ^131^I doses are needed to provide clarity on this matter.

Since 2015, many guidelines have updated the clinical application of rhTSH beyond residual ablation (13, 34, 35) in the absence of corresponding follow-up data. Our subgroup analysis of studies with a 5-year follow-up showed no significant difference between groups. As DTC can relapse 20 to 30 years after initial treatment (36, 37), patients should be observed for longer follow-up durations.

## 5 Conclusion

The cumulative data of our systematic review and meta-analysis revealed no significant difference in the medium- and long-term recurrence rate of patients receiving rhTSH or THW in preparation for RAI. More extensive RCTs, especially based on intermediate-risk and high-risk patients are needed to explore whether rhTSH can increase the risk of SIR.

## Data Availability

The datasets used and/or analyzed during the current study are available from the corresponding author on reasonable request. Requests to access these datasets should be directed to Zhongliang Wu, wzl1555@sina.com.
